# Prescription Trends and the Role of Cardiologists in the Diagnosis and Treatment of Erectile Dysfunction

**DOI:** 10.3390/jcdd12100414

**Published:** 2025-10-21

**Authors:** Filip Tkaczyk, Michal Chudzik, Aleksandra M. Piotrowska, Tim Stolpe, Julia Zurawska, Zbigniew Siudak

**Affiliations:** 1 Collegium Medicum, The Jan Kochanowski University in Kielce, 25-369 Kielce, Poland; alemarpiotrowska@gmail.com (A.M.P.); timstolpe@googlemail.com (T.S.); julia.zurawska7@gmail.com (J.Z.); zbigniew.siudak@gmail.com (Z.S.); 2 Department of Electrocardiology, Medical University of Lodz, 90-419 Lodz, Poland; michalchudzik@wp.pl

**Keywords:** erectile dysfunction, PDE-5 inhibitors, prescription patterns, cardiovascular risk

## Abstract

Introduction: Erectile dysfunction (ED) is strongly associated with metabolic and cardiovascular diseases and may serve as a marker of vascular pathology. Phosphodiesterase type 5 (PDE-5) inhibitors are the mainstay of treatment. Considering this link, cardiologists and internists might be expected to play a key role in therapy initiation. Purpose: This study evaluated prescription patterns of PDE-5 inhibitors in Poland, focusing on physician specialization and consultation type. Methods: We performed a retrospective analysis of electronic health records from over 300 outpatient clinics (2014–2024). Men >18 years with newly diagnosed ED were included. Data on the first prescriptions of sildenafil and tadalafil were assessed. Ethical approval was obtained. Results: A total of 11,998 patients were identified (mean age 42 years; mean BMI 26.54 kg/m^2^). Common comorbidities included hypercholesterolemia (67.0%), hypertension (58.5%), obesity (31.5%), and diabetes (12.4%). PDE-5 inhibitors (predominantly tadalafil (≈67%)) were prescribed in 71.5% of cases. Urologists accounted for 51.0% of prescriptions, sexologists 14.9%, internists 20.4%, and cardiologists only 0.10%. Treated patients were younger and had a lower BMI, fewer comorbidities, and more favorable metabolic profiles (all *p* < 0.05). Conclusions: Contrary to expectations, cardiologists and internists rarely initiate PDE-5 therapy. Prescribing is dominated by urologists despite high rates of cardiovascular comorbidities. A multidisciplinary approach incorporating metabolic and cardiovascular risk assessment is warranted.

## 1. Introduction

Erectile dysfunction (ED) is recognized as a multifactorial condition with biological, psychological, and social determinants. Its etiology is complex, involving both psychogenic and organic mechanisms. The disorder is strongly associated with lifestyle factors and frequently coexists with systemic diseases, particularly those of cardiovascular and metabolic origin. Among the most prevalent comorbidities are arterial hypertension, obesity, type 2 diabetes mellitus, and dyslipidemia [1,2,3,4]. Erectile dysfunction is increasingly regarded as an early clinical indicator of systemic vascular abnormalities, particularly endothelial dysfunction, leading to a reduction in nitric oxide (NO) synthesis, which results in an impairment of the vasodilation mechanism, both in the penile arteries and the coronary vessels. which may precede the onset of overt coronary artery disease [5,6,7]. From an anatomical perspective, the so-called arterial size hypothesis is crucial: because penile arteries have a smaller diameter (approximately 1–2 mm) than coronary arteries (approximately 3–4 mm), the same degree of atherosclerotic changes leads to the development of erectile dysfunction more quickly than to symptoms of coronary artery disease. In clinical practice, this translates into ED often preceding the development of clinically apparent cardiovascular disease by 2–5 years [8,9].

The mainstay of the therapeutic approach to erectile dysfunction is pharmacological treatment, which involves the individual selection of agents that influence the biochemical pathways responsible for increasing blood flow to the penis, most often phosphodiesterase type 5 (PDE-5) inhibitors, such as sildenafil and tadalafil [10,11]. In addition to improving erectile function, PDE-5s have a potential preventive effect on the cardiovascular system by improving its hemodynamics and endothelial function, reducing oxidative stress, and modulating vascular tone [12,13].

Given the close pathophysiological link between erectile dysfunction and cardiovascular disease, it can be hypothesized that cardiologists and internists will play a leading role in both the diagnosis and initiation of ED treatment. The American College of Cardiology guidelines demonstrate the strong link between erectile dysfunction, atherosclerosis, and hypertension, emphasizing the crucial role of physicians specializing in cardiovascular disease in the diagnosis and treatment of ED, particularly given the presence of comorbidities and numerous drug interactions. Experts recommend assessing cardiovascular risk in all men with ED. If risk is high, intensive interventions should be implemented, including lifestyle changes and appropriate, individualized pharmacological treatment [14]. The most reliable data on the medical specialties most frequently prescribing phosphodiesterase type 5 inhibitors (PDE5i) for erectile dysfunction come from large population-based analyses and retrospective studies. In the United States, analysis of over 100,000 cases from the MarketScan database showed that PDE-5 treatment is most frequently initiated and continued by urologists and family physicians (internists), with urologists dominating the specialist population and family physicians most frequently prescribing these medications in primary care [15]. In Europe, based on data from Italy, approximately 60% of PDE-5 inhibitor prescriptions are issued by urologists, while general practitioners (GPs) account for approximately 30%. The remaining 10% are prescriptions from other specialists, such as sexologists and cardiologists. Similar proportions are observed in Germany and the UK, where urologists remain the main prescribers, with GPs accounting for the second highest number of prescriptions [16].

The aim of this study is to assess the prescribing patterns of PDE-5 inhibitors and to evaluate the impact of physician specialization and type of consultation on the treatment of erectile dysfunction.

## 2. Materials and Methods, and Study Design

### 2.1. Study Design and Data Collection

A retrospective analysis of blinded data from electronic medical health records of patients treated in over 300 outpatient clinics by Lux Med (the largest Polish private healthcare provider) across Poland between January 2014 and December 2024 was conducted. The study was observational and retrospective, based on a cohort design, and used anonymized data collected from routine clinical practice. The study was reported in accordance with the STROBE checklist for cohort, case–control, and cross-sectional studies (combined version). Laboratory parameters included fasting glucose, total cholesterol, HDL cholesterol, LDL cholesterol (calculated by the Friedewald equation), triglycerides, thyroid-stimulating hormone (TSH), creatinine, and fasting insulin. Laboratory results were obtained from electronic medical records and interpreted according to standard clinical reference ranges: total cholesterol 120–200 mg/dL, LDL cholesterol 50–130 mg/dL, HDL cholesterol 40–100 mg/dL, triglycerides 35–150 mg/dL, fasting glucose 74–99 mg/dL, creatinine 0.6–1.3 mg/dL, TSH 0.4–4.0 mIU/L, and fasting insulin 2–25 µIU/mL. The analyzed laboratory parameters were selected due to their established role in assessing metabolic and cardiovascular risk factors, which are closely associated with the pathophysiology of erectile dysfunction.

### 2.2. Inclusion and Exclusion Criteria

Men aged ≥ 18 years who were first diagnosed with erectile dysfunction during the analyzed period were included in the statistical analysis. Exclusion criteria included: prior treatment with phosphodiesterase type 5 (PDE-5) inhibitors before the date of the first documented ED diagnosis, incomplete data on the specialization of the treating physician, lack of information on prescribed medications, and data entry errors.

Data completeness was carefully evaluated prior to statistical analysis. Core study variables—including physician specialty, type of consultation, and prescription of PDE-5 inhibitors—were available for all included participants (*n* = 11,998). Only minor missingness was observed for selected demographic and laboratory variables, reflecting the real-world structure of the dataset. Specifically, completeness was >99% for age, approximately 98% for BMI, and >95% for key laboratory parameters (cholesterol, glucose, TSH, creatinine, and insulin). Given the high overall completeness, all analyses were performed using available case data without imputation.

The study was conducted in accordance with the Declaration of Helsinki. The project was approved by the Bioethics Committee of the Jan Kochanowski University in Kielce (decision no. 5/2024). All data were anonymized, and patient identification was not possible.

### 2.3. Statistical Analysis

Normality of distributions was verified using the Shapiro–Wilk test. Variables were described using the median (Mdn) and interquartile range (IQR) for continuous data, and counts (n) and percentages (%) were provided for categorical variables. Comparisons between groups were performed using the Wilcoxon rank-sum test (for continuous variables with a nonnormal distribution) and the Pearson chi-square test or Fisher’s exact test (for categorical variables). A significance level of *p* < 0.05 was set. Analyses were performed in R (version 4.3.3) using the report (version 0.5.8), gtsummary (version 1.7.2), and dplyr (version 1.1.4) packages.

## 3. Results

### 3.1. Characteristics of the Study Sample

We analyzed the results of 11,998 adult men (age range 18–101 years) diagnosed with erectile dysfunction (ED). The study population included both younger and older patients, with a median age of 42 years (IQR: 34–52). The median BMI was 26.54 kg/m^2^ (IQR: 24.38–29.59), indicating that the study population was on the borderline between normal weight and overweight. More than two-thirds of patients met the criteria for overweight or obesity (BMI ≥ 25 kg/m^2^).

### 3.2. Assessment of the Patient Profile with ED in Relation to Comorbidities

In the analyzed group of patients with ED, the most common conditions were hypertension (58.46%) and hypercholesterolemia (66.67%), and 67.46% of the study participants had a BMI ≥ 25 kg/m^2^. Obesity was found in 31.46% of patients, and type 2 diabetes in 12.45%, confirming the dominant role of metabolic disorders in the development of erectile dysfunction. Cardiovascular diseases were also common—chronic ischemic heart disease was diagnosed in 7.10% of patients, heart failure in 2.67%, and atrial fibrillation or flutter in 3.42%. Additionally, 9.41% of patients had sleep apnea. To further characterize the study population, patients were stratified into two age groups: adults (<60 years; *n* = 10,409) and older adults (≥60 years; *n* = 1589). The median age of the entire cohort was 42 years. Older patients had a significantly higher burden of cardiometabolic comorbidities compared with younger individuals. The prevalence of hypertension was 69.8% in the ≥60 group versus 26.4% in the <60 group (*p* < 0.001), hypercholesterolemia 65.2% vs. 32.1% (*p* < 0.001), and type 2 diabetes 21.8% vs. 4.2% (*p* < 0.001). Obesity was also more common among older adults (22.3% vs. 15.8%; *p* = 0.002). The overall burden of multimorbidity was substantial, with 27.9% of patients presenting at least two comorbid conditions and 13.2% having three or more. Stratified by age, ≥2 comorbidities occurred in 21.7% of adults <60 years and in 64.8% of those ≥60 years, while ≥3 comorbidities were observed in 8.9% and 38.9%, respectively (*p* < 0.001). Spearman’s correlation analyses demonstrated a strong positive relationship between the number of comorbidities and both age (ρ = 0.49; *p* < 0.001) and BMI (ρ = 0.46; *p* < 0.001), indicating that advancing age and higher body mass index are closely associated with increased multimorbidity in men with erectile dysfunction.

### 3.3. Characteristics of Medical Consultations Among Patients with ED

The most common form of contact was an in-person visit (66.01%), and 33.99% of consultations were conducted remotely, indicating the growing importance of telemedicine. More than half of patients consulted with a urologist (50.82%), 20.39% with an internist, and 14.90% with a sexologist. However, cardiologists accounted for only 0.10% of visits, despite the high prevalence of cardiovascular disease. Between 2014 and 2024, a gradual increase in PDE-5 inhibitor prescriptions was observed, particularly for tadalafil, which became the most frequently continued therapy in recent years. Sildenafil use remained stable, while vardenafil prescriptions declined after 2019. During the COVID-19 pandemic (2020–2022), a temporary 20–30% decrease in new PDE-5 inhibitor prescriptions was noted, likely reflecting reduced in-person consultations and the shift toward telemedicine. After 2022, prescription rates gradually returned to pre-pandemic levels, indicating normalization of clinical practice.

### 3.4. Characteristics of Medications Used Among Patients with ED

Analysis of pharmacotherapy patterns in patients with ED demonstrated a widespread use of PDE-5 inhibitors, with tadalafil being the most frequently prescribed agent (64.17%), followed by sildenafil (48.47%). The preference for tadalafil may be attributed to its longer duration of action and reduced impact of food intake on efficacy. The high frequency of statin therapy, particularly rosuvastatin (17.44%) and atorvastatin (10.22%), reflects the considerable prevalence of hypercholesterolemia in the study cohort.

The notable use of antihypertensive agents, including diuretics (indapamide 10.66%, hydrochlorothiazide 8.35%) and beta-blockers (nebivolol 12.66%, bisoprolol 11.05%), further underscores the strong association between ED and cardiovascular disease, as well as the therapeutic challenges related to hypertension management. It should be noted that beta-blockers are prescribed not only for hypertension but also for other cardiovascular conditions such as coronary artery disease, heart failure, and atrial fibrillation. Because the analyzed database did not include clinical indications for each prescription, beta-blocker use may reflect treatment for various cardiovascular disorders rather than hypertension alone.

Data on the use of angiotensin-converting enzyme (ACE) inhibitors and angiotensin receptor blockers (ARBs) were not available in the database, although both classes are recommended as first-line therapy in current hypertension guidelines. The frequent prescription of nebivolol and bisoprolol suggests a clinical preference for beta-blockers with a more favorable effect on erectile function. Concomitant use of multiple cardiovascular drug classes together with PDE-5 inhibitors highlights the complexity of the clinical picture and the burden of coexisting risk factors in this population.

Detailed information on prescribed doses, duration of therapy, and treatment adjustments for PDE-5 inhibitors was not available in the analyzed database, as the records contained only data on the type of prescribed medication and the specialty of the prescribing physician. Consequently, the analysis focused on the distribution of PDE-5 inhibitor types rather than specific dosing regimens.

### 3.5. Assessment of the Association Between PDE-5 Inhibitor Use (Tadalafil, Sildenafil) and Age, BMI, and Laboratory Test Results

The comparison of demographic, anthropometric, and laboratory characteristics between patients receiving PDE-5 inhibitors and those not treated with these agents is summarized in Table 1. The data highlight significant differences in age, BMI, lipid profile, and concomitant pharmacotherapy between the groups.

### 3.6. Assessment of the Association Between Selected Medical Specialties (Urology, Sexology) and the Type of Consultation

The analysis revealed that in-office visits were the dominant model among ED patients, particularly for urology and sexology consultations. The percentage of in-office visits was significantly higher for both urologists (56.38% vs. 40.02%; *p* < 0.001) and sexologists (21.11% vs. 2.84%; *p* < 0.001) compared to out-of-office consultations. This disparity likely reflects the specific nature of ED diagnosis and treatment, which requires a physical examination, direct assessment of the genitalia, and the opportunity to build a doctor–patient relationship in an office setting, which may foster a sense of security and psychological comfort for patients.

### 3.7. Assessment of the Frequency of Specialist Visits Among Patients with ED by Physician Specialty

In the analyzed cohort, the distribution of specialist consultations is presented in Table 2. Urological (50.82%) and sexological (14.90%) visits were most frequent, clearly outnumbering consultations with internists (20.39%) and cardiologists (0.10%). This pattern likely reflects the perception of urologists and sexologists as primary specialists responsible for both the clinical assessment and therapeutic management of erectile dysfunction.

### 3.8. Assessment of the Frequency of PDE-5 Inhibitor Prescriptions in Patients with ED by Physician Specialty

Analysis of the data revealed significant differences in the frequency of PDE-5 inhibitor prescriptions between medical specialties. The majority of PDE-5 inhibitor prescriptions originated from urologists (61.71% in the PDE-5 group vs. 28.16% in the non-user group; *p* < 0.001), followed by internists (17.11%) and sexologists (11.74%), with only a marginal contribution from cardiologists (0.10%).

In the non-user group, the percentages indicate the distribution of physician specialties involved in patient consultations for erectile dysfunction, rather than the frequency of PDE-5 inhibitor prescriptions. This reflects the clinical pattern of specialist involvement even in cases where pharmacological treatment was not initiated. Collectively, urologists and sexologists were responsible for the vast majority of ED pharmacotherapy in this population, confirming their dominant role in the treatment of erectile dysfunction. Differences between these two specialties may reflect different patient profiles—including a greater proportion of psychogenic components in the sexologist group—and systemic preferences regarding medication prescription. Psychiatric consultations were rare (0.90% in the PDE-5 group) and significantly less associated with the prescription of pharmacotherapy, which resulted from the different scope of activities of this specialty in the diagnosis and treatment of ED.

### 3.9. Assessment of the Frequency of PDE-5 Inhibitor Prescriptions in Patients with ED by Type of Medical Consultation

Analysis of the frequency of PDE-5 inhibitor prescriptions by consultation method (in-person vs. remote) revealed no statistically significant differences (66.01% vs. 33.99%; *p* = 0.168). This indicates that in the studied population of men with ED, the mode of consultation itself was not a decisive factor in initiating or continuing treatment. These results may reflect a similar decision-making process among physicians in both forms of contact and simultaneously emphasize the growing role of teleconsultations as an organizationally and therapeutically equivalent alternative to in-person consultations.

## 4. Discussion

Erectile dysfunction is one of the most common health problems in men, and its prevalence increases with age and the presence of comorbidities. ED not only reduces quality of life but is also an important indicator of overall health.

Pharmacotherapy with phosphodiesterase type 5 (PDE-5) inhibitors is currently the gold standard in the treatment of erectile dysfunction, contributing to improved quality of patients’ sexual life. According to the latest Princeton IV guidelines (2024), every man with erectile dysfunction should undergo a cardiovascular risk assessment according to the ACC/AHA ASCVD 2019 algorithm, including risk factor analysis, blood pressure measurement, lipid profile, glucose level, and assessment of renal function. The guidelines indicate that ED is an early marker of atherosclerosis and endothelial dysfunction; therefore, patients with this disorder should be considered a high-risk population for whom, if in doubt, exercise testing is recommended to assess the safety of sexual activity. The importance of education, lifestyle modification, and optimal treatment of comorbidities, implemented in collaboration with cardiologists, family physicians, and nursing staff, is also emphasized [7,17]. At the same time, numerous studies indicate the need for a holistic approach, encompassing not only symptomatic therapy but also early diagnosis and regular monitoring of metabolic and cardiovascular risk factors. The type of specialist has a significant impact on the diagnosis of erectile dysfunction. In the studies by Ryan J.G. et al. and Dilixiati D. et al., family physicians and internists most often initiate the diagnosis of ED through effective risk assessment, screening, and early detection of comorbidities, in accordance with the recommendations of the American Diabetes Association [18,19]. Contrary results were obtained in our own studies, in which more than half of patients were referred to a urologist (50.8%), one in five to an internist (20.4%), and 14.9% to a sexologist; cardiologists accounted for only 0.1% of visits.

This low percentage may be explained by the fact that men with ED usually present to urologists or primary care physicians rather than cardiologists, as the condition is initially perceived as a sexual rather than cardiovascular problem. Furthermore, the absence of integrated referral pathways and limited awareness of ED as a cardiovascular risk marker contribute to the insufficient implementation of Princeton IV (2024) recommendations regarding comprehensive cardiovascular assessment and management in this patient population. Data from population-based and epidemiological studies show that fewer than one-fifth of men with newly diagnosed vascular ED are referred to a cardiologist for cardiovascular risk assessment. A retrospective Canadian study found that after initiating PDE5 inhibitor therapy, only a small percentage of patients had cholesterol (4%) or glucose (5%) screening performed, and within 90 days, there was only a moderate increase in new prescriptions for statins (10/1000 patients) and antihypertensive medications (40/1000 patients) [20]. Studies from the US and Europe indicate that most men with ED and high cardiovascular risk receive cardiology treatment (statins, beta-blockers, ACE inhibitors) primarily from general practitioners or urologists, not cardiologists. In clinical practice, fewer than 25% of patients initiate cardiovascular therapy within the first year after diagnosis [21]. This highlights the need to improve interdisciplinary collaboration and establish standardized referral pathways to cardiologists, ensuring that patients with ED and elevated cardiovascular risk receive comprehensive assessment and appropriate preventive therapy.

The results of the study by Gandaglia G. et al. emphasize that the role of cardiologists is crucial in assessing the cardiovascular risk in patients with ED, as ED may be an early indicator of coronary artery disease [22]. Initiation of therapy in patients with existing cardiovascular disease should be preceded by an individual risk analysis and clinical stabilization. The results of the studies presented by Terentes-Printzios D. et al. and Yannas D. et al. emphasize that in patients with erectile dysfunction at high cardiovascular risk, the cardiologist is responsible for intensive risk factor control and safety assessment of pharmacotherapy, including PDE-5 inhibitors, which remain contraindicated in unstable angina [23,24]. Based on the results of the studies by Jackson G. et al. and Gandaglia G. et al., the cardiological evaluation of a patient with erectile dysfunction should include a comprehensive analysis of cardiovascular risk factors, taking into account the history and physical examination (with particular emphasis on visceral obesity), the characteristics and duration of ED, laboratory tests (glycemia, lipid profile, creatinine, testosterone), ECG recording, and verification of the criteria for metabolic syndrome [17,25]. Despite the great importance in controlling cardiometabolic risk factors, internist visits were less frequent, and cardiologist consultations were exceptionally rare. The above distribution highlights a significant gap in clinical practice where patients with erectile dysfunction, often with underlying cardiovascular disease, are rarely referred for specialist cardiology evaluation.

Analyses conducted by Hackett G indicate that urologists play a crucial role in detailed diagnostics of the genitourinary system, including hormonal assessment and the use of specialized tests and therapies, especially in cases resistant to standard treatment or with complex etiologies. Psychiatrists and psychologists, in turn, are essential in the identification and treatment of erectile dysfunction resulting from depression or anxiety disorders. In our own study, (1.29%) of respondents consulted a psychiatrist [26]. Both the American Diabetes Association guidelines and the recommendations published in The New England Journal of Medicine indicate that in men with erectile dysfunction coexisting with metabolic or cardiovascular diseases, family physicians should consider PDE-5 inhibitors as first-line therapy, after excluding contraindications, especially the concomitant use of nitrates [27,28]. In a clinical approach, interdisciplinary cooperation allows for comprehensive diagnosis and treatment of erectile dysfunction, taking into account metabolic, hormonal, psychological, and cardiological aspects, which is an important condition for the effectiveness of treatment and improvement of patients’ quality of life.

In the treatment of erectile dysfunction in patients with comorbidities such as obesity, diabetes, hypertension, cardiovascular disease, chronic kidney disease, depression, or metabolic syndrome, phosphodiesterase type 5 (PDE-5) inhibitors are most commonly used, including sildenafil, tadalafil, and vardenafil; in selected cases, avanafil is also used [29]. In contrast to the results of the study by Mulhall J.P. et al., where sildenafil was the most frequently prescribed PDE-5 inhibitor, in our analysis, tadalafil dominated (64.17%), which may be related to its longer duration of action and reduced dependence on meals [15]. Tadalafil and vardenafil are widely used, especially in cases requiring a longer duration of action or a more favorable tolerability profile; however, in clinical practice, sildenafil remains the most frequently chosen drug, which is consistent with the observations by Pyrgidis N. et al. [30].

## 5. Limitations

This study has several significant limitations that should be considered when interpreting the results:

Risk of selection bias: the study population included patients from a single private healthcare facility, which may limit the generalizability of the results to other systems or populations.

Incomplete clinical data: for some patients, certain variables, such as BMI, laboratory parameters, or detailed information on comorbidities, were missing, which could have influenced the results of the statistical analyses.

Lack of long-term follow-up: it was not possible to track the effects of ED treatment over time or assess the progression of erectile dysfunction and related metabolic or cardiovascular disorders.

The database did not capture information on medication dosage or duration of therapy, and data on certain drug classes (e.g., ACE inhibitors or ARBs) were unavailable, precluding detailed comparisons with current guideline-based management.

Information on the specific clinical indications for β-blocker use was not available, which may have led to overestimation of their prescription rate for hypertension alone.

Uncontrolled confounding factors: the data did not include variables such as lifestyle, socioeconomic status, dietary habits, or treatment adherence, which could have significantly influenced the observed associations.

## 6. Implications for Future Research

Future studies on erectile dysfunction (ED) should employ a prospective, long-term follow-up design, encompassing patients from various healthcare sectors and regions of the country, to increase the representativeness of the results. Complete clinical data should be collected, including detailed metabolic and cardiovascular profiles, and lifestyle factors influencing the course of ED should be considered. Monitoring therapeutic adherence and assessing the effectiveness of integrated multidisciplinary care models in ED treatment are also crucial.

## 7. Conclusions

In the analyzed population of men with erectile dysfunction, the most common comorbidities were hypertension, hypercholesterolemia, and overweight and obesity.

PDE-5 inhibitor therapy was significantly more frequently initiated by urologists and sexologists than by other specialists; the form of consultation (in-person vs. remote) did not significantly influence the frequency of prescription of these medications. This indicates the need for broader involvement of cardiologists in multidisciplinary care for patients with ED, both in terms of diagnosis and the prevention of cardiovascular complications.

The obtained results emphasize the need for an integrated, holistic approach to the treatment of erectile dysfunction, taking into account vascular, metabolic, and psychogenic aspects, with an emphasis on modifying risk factors.

## Figures and Tables

**Table 1:** Comparison of demographic, anthropometric, laboratory, and pharmacotherapy characteristics between patients with erectile dysfunction treated and not treated with PDE-5 inhibitors.

Characteristic	Use of PDE-5 Inhibitors		*p* ^c^
	Yes, *n*_1_ = 7851 ^a^	No, *n*_2_ = 721 ^a^	
Age, years	45.00 (36.00, 55.00)	47.00 (40.00, 56.00)	<0.001
BMI, kg/m^2^	26.87 (24.69, 29.94)	27.78 (25.18, 31.14)	<0.001
LDL cholesterol, calculated	115.80 (91.20, 141.40)	119.50 (93.84, 150.43)	0.019
LDL cholesterol > 135	1019 (30.37%) ^b^	144 (36.73%) ^b^	0.010 ^d^
Cholesterol	190.25 (163.00, 220.00)	199.40 (165.00, 233.35)	<0.001
Cholesterol HDL	47.70 (40.00, 56.00)	45.00 (39.00, 53.00)	0.002
Cholesterol no-HDL	137.86 (109.00, 170.00)	155.55 (117.85, 182.75)	<0.001
Triglycerides	112.20 (77.00, 161.95)	130.00 (89.00, 198.98)	<0.001
Glucose	91.20 (86.00, 98.00)	92.70 (86.80, 99.71)	0.010
Insulin	12.50 (7.82, 19.63)	12.90 (7.80, 18.30)	0.716
Creatinine	0.96 (0.87, 1.07)	0.96 (0.87, 1.05)	0.477
TSH	1.66 (1.18, 2.33)	1.82 (1.25, 2.35)	0.053
TSH normal	3121 (96.57%) ^b^	341 (96.33%) ^b^	0.816 ^d^
HMG-CoA reductase inhibitors	1667 (21.23%)	389 (53.95%)	<0.001 ^d^
Selective beta-blockers—beta-1 receptor blockers	1415 (18.02%)	361 (50.07%)	<0.001 ^d^
Thiazide diuretics	589 (7.50%)	127 (17.61%)	<0.001 ^d^
Obesity	1391 (17.72%) ^b^	224 (31.07%) ^b^	<0.001 ^d^
Arterial hypertension	2799 (35.65%) ^b^	552 (76.56%) ^b^	<0.001 ^d^
Chronic ischemic heart disease	372 (4.74%) ^b^	58 (8.04%) ^b^	<0.001 ^d^
Heart failure	132 (1.68%) ^b^	32 (4.44%) ^b^	<0.001 ^d^
Atrial fibrillation and flutter	170 (2.17%) ^b^	39 (5.41%) ^b^	<0.001 ^d^
Non-insulin-dependent diabetes	624 (7.95%) ^b^	109 (15.12%) ^b^	0.628 ^d^
Insulin-dependent diabetes	189 (2.41%) ^b^	20 (2.77%) ^b^	<0.001 ^d^
Hypercholesterolemia	3120 (39.74%) ^b^	501 (69.49%) ^b^	<0.001 ^d^
Sleep apnea	418 (5.32%) ^b^	69 (9.57%) ^b^	<0.001 ^d^
Sleep onset and sleep maintenance disorders	117 (1.49%) ^b^	16 (2.22%) ^b^	0.174 ^d^

^a^ Mdn (IQR); ^b^ n (%); ^c^ Wilcoxon rank sum test; ^d^ Pearson’s chi-square test. Data are presented as median (interquartile range, IQR) for continuous variables and as number (percentage) for categorical variables. Columns 3 and 4 correspond to patients treated with PDE-5 inhibitors (*n*_1_ = 7851) and those not treated with PDE-5 inhibitors (*n*_2_ = 721), respectively. Statistical comparisons between groups were performed using the Wilcoxon rank-sum test for continuous variables and Pearson’s chi-square test for categorical variables.

**Table 2:** Distribution of specialist consultations in patients with erectile dysfunction.

Characteristics	*N*	*n* (%)
Type of Consultation	11,998	
In-person		7920 (66.01%)
Teleconsultation		4078 (33.99%)
Urologist	11,998	6099 (50.82%)
Sexologist	11,998	1788 (14.90%)
Internist	11,998	2446 (20.39%)
Cardiologist	11,998	12 (0.10%)
Psychiatrist	11,998	155 (1.29%)
Endocrinologist	11,998	929 (7.74%)
Other Specialties	11,998	571 (4.76%)

## Data Availability

The datasets generated and analyzed during the current study are not publicly available due to data protection regulations. Reasonable requests for access to anonymized data may be directed to the corresponding author.
